# Inferring Phylogenies from RAD Sequence Data

**DOI:** 10.1371/journal.pone.0033394

**Published:** 2012-04-06

**Authors:** Benjamin E. R. Rubin, Richard H. Ree, Corrie S. Moreau

**Affiliations:** 1 Committee on Evolutionary Biology, University of Chicago, Chicago, Illinois, United States of America; 2 Department of Zoology, Field Museum of Natural History, Chicago, Illinois, United States of America; 3 Department of Botany, Field Museum of Natural History, Chicago, Illinois, United States of America; Barnard College, Columbia University, United States of America

## Abstract

Reduced-representation genome sequencing represents a new source of data for systematics, and its potential utility in interspecific phylogeny reconstruction has not yet been explored. One approach that seems especially promising is the use of inexpensive short-read technologies (e.g., Illumina, SOLiD) to sequence restriction-site associated DNA (RAD) – the regions of the genome that flank the recognition sites of restriction enzymes. In this study, we simulated the collection of RAD sequences from sequenced genomes of different taxa (*Drosophila*, mammals, and yeasts) and developed a proof-of-concept workflow to test whether informative data could be extracted and used to accurately reconstruct “known” phylogenies of species within each group. The workflow consists of three basic steps: first, sequences are clustered by similarity to estimate orthology; second, clusters are filtered by taxonomic coverage; and third, they are aligned and concatenated for “total evidence” phylogenetic analysis. We evaluated the performance of clustering and filtering parameters by comparing the resulting topologies with well-supported reference trees and we were able to identify conditions under which the reference tree was inferred with high support. For *Drosophila*, whole genome alignments allowed us to directly evaluate which parameters most consistently recovered orthologous sequences. For the parameter ranges explored, we recovered the best results at the low ends of sequence similarity and taxonomic representation of loci; these generated the largest supermatrices with the highest proportion of missing data. Applications of the method to mammals and yeasts were less successful, which we suggest may be due partly to their much deeper evolutionary divergence times compared to *Drosophila* (crown ages of approximately 100 and 300 versus 60 Mya, respectively). RAD sequences thus appear to hold promise for reconstructing phylogenetic relationships in younger clades in which sufficient numbers of orthologous restriction sites are retained across species.

## Introduction

In the practice of molecular systematics, a common goal is to efficiently sample as much informative data from the genomes of as many taxa as possible for phylogeny reconstruction. For many years, pursuit of this goal has emphasized sequencing orthologous genes, with individual studies typically sampling few genes relative to the number of taxa. This low gene-to-taxon ratio is due largely to the painstaking effort often required to find genes that can both be reliably amplified and sequenced across the taxa of interest, and have sufficient variation to confidently resolve phylogenetic relationships. In some clades, comparative genomics has led to substantial increases in the number of candidate genes available for screening (e.g., COS, COSII; [1], [2]), and some recent phylogenetic studies have notably sampled many more genes than before (e.g. [3]–[7]). However, widespread sampling of the genome generally remains difficult and rare outside of model taxa.

In contrast to the traditional gene-centric approach, second generation short-read sequencing technology (e.g. Illumina and SOLiD) offers an alternative method of sampling genome-wide nucleotide variation in the form of restriction site associated DNA (RAD) sequencing [8], which targets the flanking regions of restriction sites. RAD was initially developed for large-scale microarray-based genotyping in two model organisms, the threespine stickleback (*Gasterosteus aculeatus*) and the fungus *Neurospora crassa* [9]–[11]. Baird and colleagues [8] were the first to combine RAD with the Illumina short-read sequencing platform, and described a new method for multiplexed sequencing of the flanking regions of restriction sites with high coverage, which they used to find more than 13,000 single nucleotide polymorphisms (SNPs) and map traits in the threespine stickleback and *N. crassa*.

By using restriction sites to reduce genomic representation, RAD sequencing preferentially targets orthologous regions (to the extent that restrictions sites are conserved across individuals) that are scattered throughout the genome. As such, it potentially represents a more cost-effective means of generating comparative genomic data for molecular systematics than sequencing and assembling entire genomes at the same level of coverage. RAD sequencing has only recently begun to be applied to studies of non-model organisms and/or natural populations, and to date these have only addressed questions at or below the level of a single species. For example, Emerson et al. [12] used RAD sequencing to identify over 3,700 SNPs in a phylogeographic study of the pitcher plant mosquito (*Wyeomyia smithii*), and Hohenlohe et al. [13] used the method to conduct population genomic analysis of natural populations of threespine stickleback (*G. aculeatus*).

Can RAD sequences be advantageously applied to interspecific phylogeny reconstruction in the absence of a reference genome? The challenge presents a number of potential problems, such as: the orthology relationships among sequences are unknown at the outset, and must be estimated; evolution of restriction sites is expected to yield missing data (incomplete samples of orthologous sequences across taxa); and the genetic linkage relationships among loci are unknown. In this paper, we describe a simple workflow for processing RAD sequences and assembling data matrices for “total evidence” phylogenetic analysis, and conduct a series of *in silico* experiments that test its performance in the context of sequenced genomes and known relationships within *Drosophila*, mammals, and yeasts, which represent a range of genome sizes and evolutionary divergence times. We focus specifically on two key questions: (1) In the absence of a reference genome, can the orthology of RAD sequences be accurately assessed across species? (2) What parameters for assembling phylogenetic data matrices yield the most accurate and well-supported trees?

## Methods

### Simulating RAD sequencing using known genomes

We generated RAD data sets by scanning fully sequenced genomes of *Drosophila* (12 species), mammals (11 species), and yeasts (*Saccharomyces*: seven species, and one outgroup species, *Candida albicans*). The *Drosophila* genomes were downloaded together as an alignment from http://www.biostat.wisc.edu/~cdewey/fly_CAF1/data/fly_CAF1.1.tar.gz. All mammal genomes were downloaded individually from the NCBI genome resources server (http://www.ncbi.nlm.nih.gov/genome/guide/) as contigs or assembled chromosomes if available. Yeast genomes were downloaded from GenBank under accession numbers: *S. cerevisiae*, BK06935–BK06949; *S. paradoxus*, AABY01000000; *S. mikatae*, AABZ01000000; *S. bayanus*, AACA01000000; *S. kluyveri*, AACE00000000; *S. castellii*, AACF00000000; *S. kudriavzevii*, AACI00000000; *C. albicans*, AACQ01000000. All species sampled are listed with their genome sizes in Table 1.

**Table 1:** Species of *Drosophila*, mammals, and fungi included in this study.

Species	Genome size (bases)	#50 bp reads	
*Drosophila*		*NotI*	*SbfI*
*D. ananassae*	151452809	3628	5230
*D. erecta*	125196136	3072	4372
*D. grimshawi*	136438528	2166	2224
*D. melanogaster*	120416594	2666	4000
*D. mojavensis*	156491326	4034	3062
*D. persimilis*	144081254	4636	5146
*D. pseudoobscura*	136104117	4704	5114
*D. sechellia*	120266937	2698	4208
*D. simulans*	125504156	2672	4058
*D. virilis*	155510099	4960	3176
*D. willistoni*	171979099	1540	2038
*D. yakuba*	123618427	2866	4288

Mammals		*NotI*	
*Bos taurus*	2906457325	19096	
*Canis lupus familiaris*	2418212456	27736	
*Cavia porcellus*	1949659178	15584	
*Equus caballus*	2474878062	16850	
*Homo sampiens*	2775440241	16496	
*Macaca mulatta*	3011952279	17440	
*Mus musculus*	2593552788	11930	
*Oryctolagus cuniculus*	2076044328	32996	
*Pan troglodytes*	3010437433	17406	
*Pongo pygmaeus*	3092841440	17168	
*Rattus norvegicus*	2663612739	8890	

Fungi		*EcoRI*	
*Saccharomyces bayanus*	11865314	8608	
*S. castellii*	11242286	9618	
*S. cerevisiae*	11122836	8068	
*S. kluyveri*	11506563	7028	
*S. kudriavzevii*	11177778	8630	
*S mikatae*	10772608	7818	
*S. paradoxus*	11872617	8870	
*Candida albicans*	14284095	10094	

Genome size is presented in the total number of nucleotides (bases). #50 bp reads is the number of simulated RAD sequences using the given restriction enzyme. #100 bp reads was similar but could differ slightly because we did not include sequences that failed the length requirement.

For each genome, we simulated RAD data by sampling either short (50 base pair (bp)) or long (100 bp) sequences immediately upstream and downstream of all restriction sites of the enzymes *SbfI* (5′-CCTGCAGG-3′), *NotI* (5′-GCGGCCGC-3′), or *EcoRI* (5′-GAATTC-3′). We chose 50 and 100 bp because these are typical read lengths currently produced by the Illumina sequencing platform. Restriction site sequences themselves were not included in the simulated reads. Restriction enzymes vary in cut frequency and hence in the number of RAD sequences generated, and our goal was to harvest datasets of manageable size (based on preliminary studies), i.e., on the order of no more than a few million sequences in total from each group (*Drosophila*, mammals, and yeasts). For *Drosophila*, we chose the enzymes *SbfI* and *NotI* because they recognize longer, GC rich sequences that occur less frequently across the genome. We also recorded the position of each sequence in the original alignment to facilitate assessment of the accuracy of orthology estimation. Mammal genomes are relatively large, so we used only the most selective restriction enzyme, *NotI*, while for the smaller yeast genomes we chose the more frequent cutter, *EcoRI*.

In practice, RAD sequencing yields millions of Illumina reads that must be processed into consensus sequences at each locus for each sample, accounting for sequencing error and variation (e.g., heterozygosity). Methods for doing so have been explored by Li et al. [14], Emerson et al. [12], Hohenlohe et al. [13], and Catchen et al. [15]. In this study, we assume that the data have been processed to this point.

### A phylogenetic workflow for RAD sequence data

For each data set, our goals were to cluster and align orthologous sequences across species, concatenate alignments into a character matrix, reconstruct the phylogeny, and measure its effectiveness at correctly inferring evolutionary relationships. Our strategy for finding phylogenetically informative loci is based on initially clustering the data by sequence similarity and then filtering the resulting clusters by taxonomic coverage.

#### 1. Clustering

In the absence of a reference genome, sequence similarity is the simplest way to infer orthology. We clustered each data set by sequence similarity using UCLUST v2.0 [16]. The UCLUST algorithm facilitates rapid clustering of large data sets by avoiding exhaustive pairwise comparisons, instead heuristically using representative seed sequences that are typically the longest sequences in the data set (in our case, RAD sequences are all of equal length and seeds are created from the first sequences in the input file that do not match the previous seeds). It produces clusters in which the identity of each sequence to the seed is equal to or greater than the specified similarity value. Seeds are created as the sequence input file is traversed, meaning that results are potentially dependent on input order. To account for this effect, we randomized the order of sequences and repeated clustering and all subsequent steps in the analysis five times for each *Drosophila* data set. We calculated the means and standard errors of the characteristics and accuracies of trees resulting from these replicates for each set of parameters. For a subset of these data sets, we then performed 100 additional randomizations of sequence input order and subsequent phylogenetic analyses to more thoroughly estimate the distribution of phylogenetic results arising from variation in sequence input order.

We tested a range of similarity values for clustering: for *Drosophila*, 50–95% in 5% increments; for mammals, 55, 70, and 90%; and for yeasts, 50–90% similarity in 10% increments. Fewer similarity values were explored for mammals and yeasts due to the larger sizes of those data sets and the computation time required for each parameter investigated. All other UCLUST parameters were left at their default values.

#### 2. Filtering

Clustering by sequence similarity is clearly an imperfect solution to the problem of orthology assessment. An ideal cluster in this context would contain a single orthologous sequence from each taxon in the data set. However, sequences may be similar but not orthologous, e.g., in the case of duplicated genes or repetitive elements, or orthologous but not similar, e.g. due to evolutionary divergence. For these reasons some clusters are expected to contain more than one sequence per taxon, and/or fewer than the total number of taxa. We therefore filtered clusters in two sequential steps: we first discarded all clusters containing more than one sequence from a single species, then discarded those clusters with fewer taxa than a specified minimum threshold number (a parameter we refer to hereafter as “min. taxa”). The minimum number of taxa required for an informative unrooted phylogenetic tree is four. In the interest of understanding how the proportion of missing data affects phylogenetic accuracy, for the latter step we tested min. taxa values of four, six, and nine for *Drosophila*, four and six for mammals, and four and five for yeasts.

#### 3. Alignment, supermatrix assembly, and tree inference

For each data set and parameter combination, filtered clusters were individually aligned using MUSCLE v3.8 [17] and concatenated into a single total evidence supermatrix [18], with missing data symbols inserted as needed. This yielded 600 supermatrices for Drosophila (i.e., from five input-order replicates of 120 combinations of clustering and filtering parameters), 20 for mammals, and 20 for yeasts. For each Drosophila supermatrix, we identified clusters that consisted entirely of orthologous sequences from the reference genome alignment, and created two submatrices for separate analysis: one containing only orthologous clusters, and one of only non-orthologous clusters. For each supermatrix, a maximum likelihood tree was inferred using RAxML v7.0.4 [19] under the general time-reversible nucleotide model with gamma-distributed rate heterogeneity and invariant sites (GTRGAMMAI).

### Benchmark RAD trees

To assess the impact of orthology estimation errors on phylogenetic inference, we conducted a series of “best-case” benchmark analyses in which orthology was known *a priori*. We scanned the *Drosophila* genome alignment for restriction sites, and extracted subalignments of orthologous RAD sequences for each restriction site. In each subalignment, taxa for which the restriction site was missing (due to nucleotide variation disrupting the recognition sequence or the loss of the entire locus) were excluded, and missing data symbols were inserted in their place to simulate real data. Subalignments were then concatenated and subjected to phylogenetic analysis as described above. The procedure was repeated with the same combinations of restriction enzyme, read length, and min. taxa from steps 1 and 2 (Table S1), resulting in 12 matrices of known orthology (two read lengths×two restriction enzymes×three min. taxa sizes).

### Measuring phylogenetic accuracy

**Figure 1:**
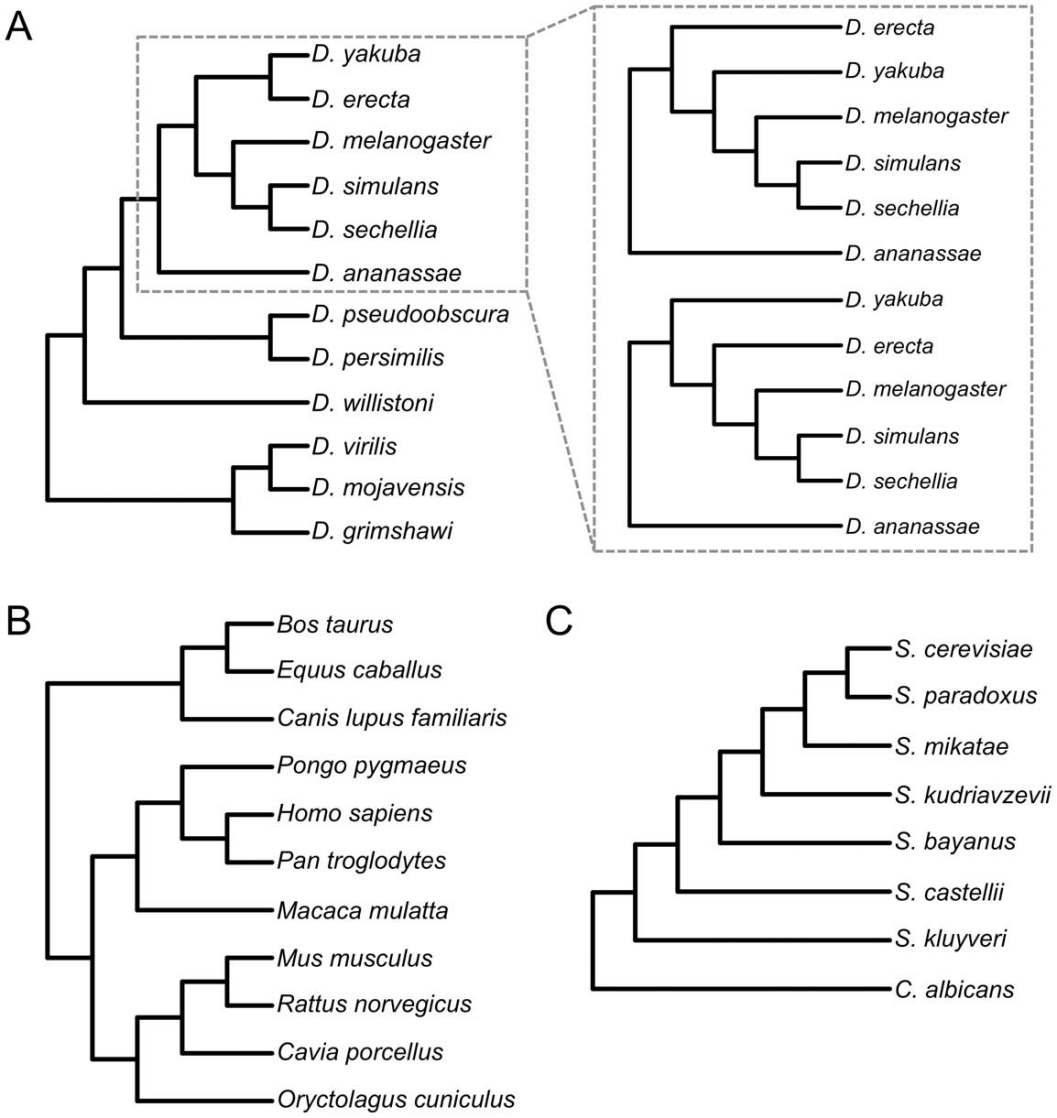
Reference phylogenies of each study group. All branch lengths are arbitrary and do not indicate evolutionary distance. A) *Drosophila* phylogeny modified from [4]. The inset shows the two alternative topologies commonly supported by individual gene trees in [32]. B) Reference mammal phylogeny from [20]. C) Reference yeast phylogeny from [3].

Phylogenetic accuracy was evaluated by comparing inferred trees with published reference phylogenies (Fig. 1). The reference phylogeny for *Drosophila*, from [4], was estimated using neighbor-joining, parsimony, and Bayesian inference, and all of the nodes have bootstrap and posterior probability support values at or close to 100%. The reference phylogeny for mammals [20] is based on maximum likelihood and Bayesian inference of coding sequences orthologous to 10 genes in a 1.9 Mb region of human chromosome 7 using RY-coding (i.e. only transversions were used for inference) to reduce non-phylogenetic signal. All but a single node have 100% posterior support (the node which places human and chimpanzee as sister species has 36% posterior support) and this is due to insufficient signal (low number of transversions) between primate sequences [20]. When transitions were included in the analysis, this node was also highly supported (100% posterior support). The reference phylogeny for yeast [3] is based on maximum likelihood and maximum parsimony analyses of a concatenated matrix of 106 genes, and all nodes are strongly supported with bootstrap values of 100%.

We quantified phylogenetic accuracy by counting the number of correct nodes on the inferred trees. A node was counted as correct if it defined a taxon bipartition that was identical to one present in the reference phylogeny. Node support was measured from 100 nonparametric bootstrap replicates in RAxML for each supermatrix. We compared bootstrap values of correct and incorrect nodes over sets of trees using Mann-Whitney U tests due to the non-independence of each tree.

### Multilocus species tree inference

The large number of loci produced by RAD sequencing motivate the question of whether the data can be used in the context of species tree estimation methods that apply coalescent theory to multiple unlinked loci, and infer a tree while accounting for incomplete lineage sorting of individual genes (e.g. [21]–[23]). We proceeded under the assumption that each locus was unlinked, to simulate conditions in which no reference genome is available. We used *BEAST [22] to estimate the species tree of *Drosophila*. Due to limits of computer memory, we analyzed only a subset of 20 parameter combinations with the smallest data matrices (Table S2). All analyses assumed unlinked substitution models and tree models, constant population size, a strict molecular clock, a general time-reversible substitution model with gamma-distributed rate heterogeneity and invariant sites with estimated base frequencies, a birth death prior for trees, and a birth death prior for the species tree. Each was run for 10 million iterations, sampling every 1,000 iterations with a burn-in of 1,000 trees.

## Results

### Simulated RAD sequencing

The number of RAD sequences obtained from each species and restriction enzyme are summarized in Table 1. The number of RAD site sequences (RAD loci) harvested per species per read length was: 1,500–5,300 (*Drosophila*), 9,000–33,000 (mammals), and 7,000–10,000 (yeasts). These matrices ranged in total length from 100 bp to 538,058 bp.

### Orthology estimation

**Figure 2:**
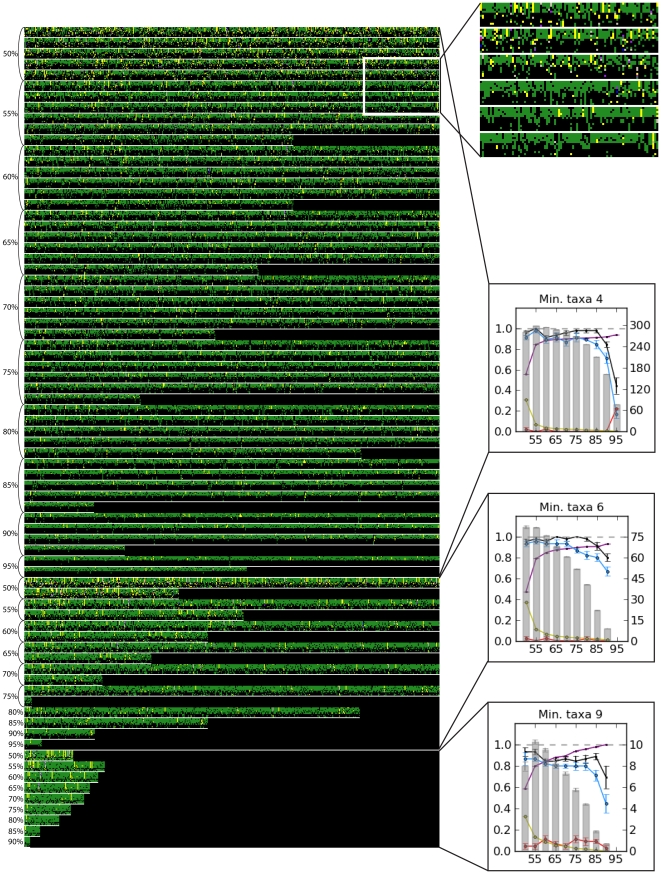
The orthology of one replicate of the 100 bp *SbfI Drosophila* matrices based on the concatenated alignment (701-299,470 bp) of all 12 genomes after restriction cutting and clustering without prior knowledge of orthology. Each column of square pixels bounded by white lines represents a single cluster (locus) produced by a given set of parameters. Each row within these clusters represents a single taxon. Therefore, between each pair of horizontal white lines is a grid where rows are taxa and columns are clusters. The order of taxa from top to bottom of each cluster is: *D. simulans*, *D. sechellia*, *D. melanogaster*, *D. yakuba*, *D. erecta*, *D. ananassae*, *D. pseudoobscura*, *D. persimilis*, *D. willistoni*, *D. virilis*, *D. mojavensis*, and *D. grimshawi*. The area in the white box is blown up in the inset to show detail. Within a cluster, black indicates that a taxon did not have a sequence in that cluster. Colors in a cluster represent orthologous sequences. For example, the top right cluster (or last column in the top row) in the expanded portion contains orthologous sequences from *D. simulans* and *D. sechellia* (yellow), and orthologous sequences from *D. melanogaster*, *D. yakuba*, and *D. erecta* (green), though sequences from the two groups are not orthologous. The cluster immediately to the left contains orthologous sequences from *D.pseudoobscura*, *D. persimilis*, *D. mojavensis*, and *D. grimshawi*. The values of similarity used for clustering the sequences in each matrix are indicated on the left and the minimum threshold number of taxa (min. taxa) is indicated by the plots on the right. These plots are exactly as in Fig. 3. Note that many parameter combinations yield matrices that span several lines. The boundaries between matrix representations are indicated on the left.

The performance of UCLUST in clustering orthologous sequences of *Drosophila*, as measured by the proportion of clusters consisting entirely of orthologs from the aligned reference genomes, is summarized in Table S2. Predictably, higher similarity values in clustering yielded better recovery of orthologous sequences. At the lowest value of similarity (50%), the proportion of orthologous clusters ranged from 0% (restriction enzyme = *NotI*, read length = 50 bp, min. taxa = 9) to 60% (restriction enzyme = *SbfI*, read length = 100 bp, min. taxa = 9), with a mean of 27%. For the highest value of similarity (95%), the mean percentage of clusters that were completely orthologous was 87%. Figs. 2, S1, S2, and S3 show patterns of orthology in the complete concatenated data matrix for one replicate of clustering from each parameter set.

Across all parameter combinations, the average proportion of completely orthologous clusters for which the MUSCLE alignment of the RAD clusters matched the original genome alignment was 45%. This ratio ranged from 0–100% and was generally higher at higher clustering similarities (10% for matrices with cluster similarity = 50% and 66% for matrices with cluster similarity = 95%).

### Filtering clusters by taxonomic coverage

Removal of clusters with more than one sequence per taxon, and with fewer than a threshold number of taxa (min. taxa), yielded data sets that varied in total aligned length and in the proportion of missing data. For *Drosophila*, matrices ranged in total length from 100 bp (restriction enzyme = *SbfI*, read length = 50 bp, min. taxa = 9, cluster similarity = 90 and 95%; only two loci met this stringent criterion) to 299,761 bp (restriction enzyme = *SbfI*, read length = 100 bp, min. taxa = 4, cluster similarity = 55%; 2,844 RAD loci; Table S2). The proportion of missing data ranged from 6%–67% (Table S2). Applying higher values of clustering similarity and minimum taxa tended to reduce the total proportion of missing data, but decreased the overall concatenated matrix length. For mammals, concatenated alignments ranged in length from 26,840 bp (read length = 100 bp, min. taxa = 6, cluster similarity = 90%; 266 RAD loci) to 538,058 bp (read length = 100 bp, min. taxa 4, cluster similarity = 55%; 4,916 RAD loci) with between 38% and 59% missing data (Table S3). Lengths of concatenated alignments for yeast ranged from 1,063 bp (read length = 50 bp, min. taxa = 5, cluster similarity = 90%; 20 RAD loci) to 111,721 bp (read length = 100 bp, min. taxa = 4, cluster similarity = 50%; 935 RAD loci) with 21–38% missing data (Table S4).

Certain parameter sets produced incomplete matrices, in which data were entirely lacking for one or more taxa. For *Drosophila*, these conditions were: read length = 100 bp and cluster similarity = 90% or 95%, for all values of min. taxa (15 incomplete matrices using min. taxa = 9; 9 using min. taxa = 6; and 4 using min. taxa = 4). In general, incomplete matrices were more likely using high values of both clustering similarity and min. taxa (Table S2). We did not infer trees in these cases. Figure 2 shows patterns of missing data in one set of *Drosophila* clusters obtained using 100 bp reads, restriction enzyme *SbfI*, and all min. taxa used; patterns for other combinations of sequence length and restriction enzyme are shown in Figures S1, S2, and S3. For yeasts, the parameter sets that led to incomplete matrices were as follows: read length = 100 bp, min. taxa = 5, clustering similarity = 90%; read length = 50 bp, min. taxa = 5, clustering similarity = 70%, 80%, and 90%.

Sequence input order had little effect on the number of clusters that were assembled into a supermatrix for a given analysis. Randomized replicates of a given parameter set yielded differences in cluster number ≤98 (out of approximately 2,500 clusters; 3.8% of maximum number of clusters; restriction enzyme = *SbfI*, 100 bp reads, min. taxa = 4, cluster similarity = 50%). Within each set of five replicates, the proportional difference of the number of clusters between replicates (1 – *n*/*m*, where *n* is the minimum number of clusters and *m* is the maximum number) was always less than 6% with min. taxa = 4. For all parameter combinations with read length = 100 bp, the proportional difference of clusters was 25% or less. The largest proportional difference was 66% (restriction enzyme = *NotI*, 50 bp reads, min. taxa = 9, cluster similarity = 50%). In general, more moderate values of sequence similarity (55%–85%) produced less variation in the number of clusters across replicates.

Across all parameter combinations, invariant clusters (i.e., composed of identical sequences) were rare, never exceeding 3.5% of all clusters with read length = 100 bp, and 16% of all clusters with read length = 50 bp (Table S2). However, the number of variable sites within clusters did decrease with increasing values of cluster similarity.

### Phylogenetic accuracy

#### *Drosophila*

The benchmark maximum likelihood trees for *Drosophila* reconstructed from data sets of known orthology and alignment (12 matrices) were topologically accurate for all nodes with only one exception: the analysis using restriction enzyme *NotI*, read length = 50 bp, and min. taxa = 4 had one incorrect node (*Drosophila virilis* sister to *D. grimshawi* instead of *D. mojavensis*), but this clade received little bootstrap support (26%). The concatenated alignments ranged from 2,700 bp to 259,000 bp in length, containing 681 to 46,342 variable sites and 380 to 16,836 parsimony informative sites, respectively. At least eight out of the nine nodes in every tree were supported by bootstrap values greater than 70%. Benchmark trees reconstructed from *SbfI* datasets were topologically accurate and had high support at all nine nodes (Table S1).

**Figure 3:**
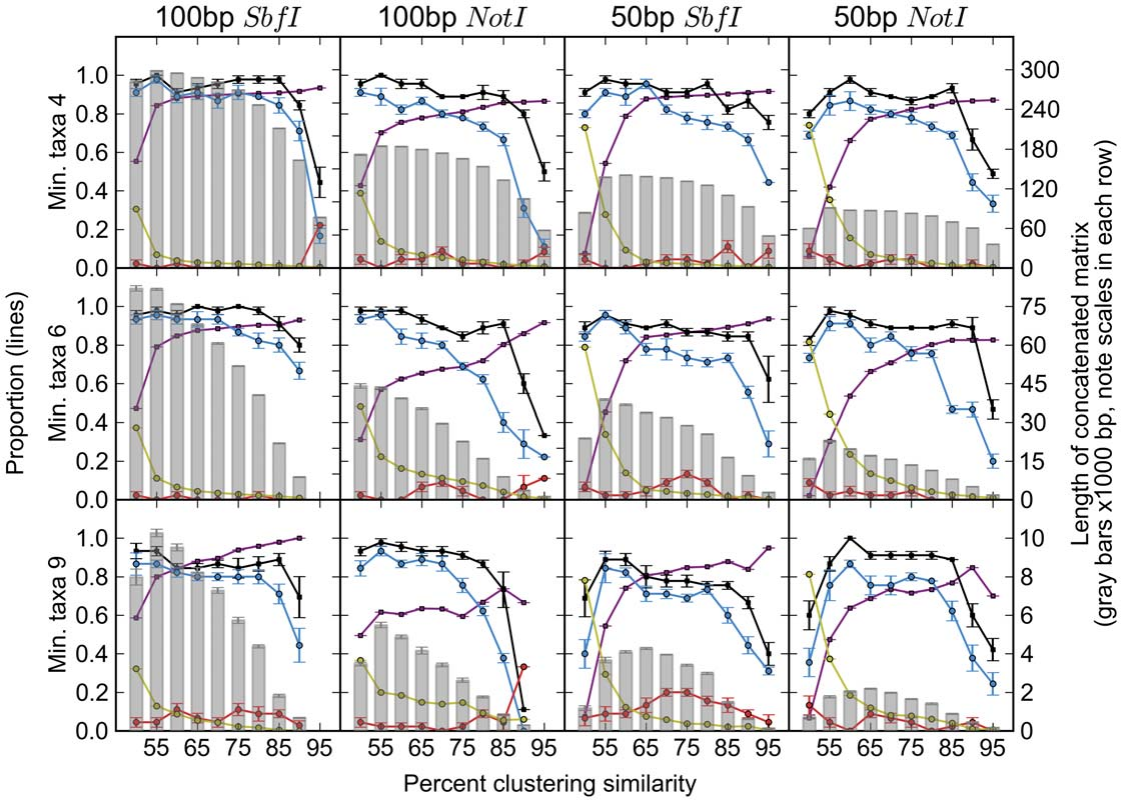
Accuracy of the RAD method for inferring *Drosophila* phylogeny. Proportions are indicated on the left axis. The x-axis shows the percent similarity used for clustering, the three rows show each minimum cluster size, and the read lengths and restriction sites used are indicated by column. Gray bars represent total matrix length as represented on the right axis. Black points are the mean proportion of correct nodes in a tree (out of a total of 9), blue points are the mean proportion of correct nodes with bootstrap support greater than 70%, and red points are the mean proportion of incorrect nodes with bootstrap support greater than 70%. Purple points are the proportion of clusters that are orthologous and yellow points are the proportion of invariant sites within clusters. Results from every set of parameters are shown. Points represent the mean ± SE of the five replicates of clustering, filtering, and tree inference for each set of parameters with randomized input order of sequences into UCLUST. However, not all parameters produced five usable matrices (one or more taxa with all empty sequence). The number of successful replicates is shown in Table S2.

For data sets in which orthology was estimated, topological accuracy varied according to which clustering and filtering parameters were used. Figure 3 summarizes these results. In general, longer reads and lower values of clustering similarity and minimum taxa yielded larger concatenated matrices and more accurate topologies. Out of 572 phylogenetic analyses (120 parameter combinations each replicated five times, excluding 28 replicates yielding incomplete matrices), 195 (34%) produced completely accurate topologies, and 263 (46%) produced topologies with only one incorrect node (of nine total).

Matrices consisting of only orthologous clusters yielded phylogenies as accurate as full matrices in 345 cases, more accurate in 166 cases, and less accurate in 61 cases. Non-orthologous matrices produced phylogenies that were more as accurate as the full matrices 161 times, more accurate in 52 cases, and less accurate in 359 cases (Table S2).

Bootstrap support was not a consistent indicator of node accuracy (Fig. 3). Our analyses of *Drosophila* yielded 572 trees, representing 5,148 nodes. Most of these nodes (4,446; 86%) were correct and of these, 3,758 (85%) were supported with bootstrap values of 70% or greater. Among the 702 incorrect nodes, 218 (31%) were supported with bootstrap values of 70% or greater. Overall, the mean bootstrap support was 50% for incorrect nodes and 88% for correct nodes. This difference was highly significant (Mann-Whitney test: *U* = 2.2×10^7^, *P*<2.2×10^−16^).

We similarly compared bootstrap support between correct and incorrect nodes across all trees based on each individual parameter (17 subsets of trees: two for restriction enzymes, two for read lengths, three for min. taxa, and 10 for clustering similarities; Table S5). In these analyses, two nodes in the *Drosophila* phylogeny often did not have significantly higher bootstrap support than incorrect nodes (Node 3 and Node 9 in Table S5). The support for one of these nodes (ingroup taxa: *D. yakuba* and *D. erecta*; Node 3 in Table S5) was not significantly higher than the support for all incorrect nodes for 13 subsets of trees and was significantly lower than incorrect node support in one case (trees with clustering similarity = 75%). The second node that often had low support (ingroup taxa: *D. virilis* and *D. mojavensis*; Node 9 in Table S5) had significantly lower support than the incorrect nodes among trees with clustering similarities = 80% and 85%. Support for this node was not significantly different than support for incorrect nodes in six additional cases. The results of all of these tests are shown in Table S5. Although most correct nodes are better supported on average than incorrect nodes, 41 incorrect nodes had 100% bootstrap support.

The amount of missing data varied across parameter combinations but *D. willistoni*, *D. grimshawi*, *D. virilis*, and *D. mojavensis* all had more missing data than the eight other taxa in 518 of 572 matrices (91%). These taxa were also represented by fewer parsimony informative sites than all other taxa in 558 of 572 matrices (98%). The lack of phylogenetically informative data may explain cases of topological inaccuracies and low bootstrap support within the clade containing *D. grimshawi*, *D. virilis*, and *D. mojavensis* (Fig. 1; Table S2).

Sequence input order had a detectable effect on phylogeny reconstruction, with randomized replicates producing different results in some cases. Only 13 (11%) of the 120 parameter combinations produced exactly the same topology in all replicates (N = 5). However, 49 (41%) of the 120 parameter combinations yielded topologies that, among the five replicates, were either completely correct or incorrect at only a single node.

For all clustering similarities and two sets of read length, restriction enzyme, and minimum taxa (restriction enzyme = *SbfI*, 100 bp reads, min. taxa = 4 and restriction enzyme = *NotI*, 50 bp reads, min. taxa = 9) we performed 100 additional randomized replicates (Table S6). The phylogenies resulting from each parameter set tended to converge to a particular level of accuracy with this number of replicates. Combinations with restriction enzyme = *SbfI*, 100 bp reads, and min. taxa = 4 tended to converge to perfect topologies, and combinations with restriction enzyme = *NotI*, 50 bp reads, and min. taxa = 9 converged to eight correct nodes (out of nine). Those parameter combinations in the subset tested that yielded less correct phylogenies produced wider distributions of phylogenetic accuracy (Table S6).

#### Mammals and yeasts

For mammals, all trees had a minimum of four correct nodes (out of 8 total) but none completely matched the reference topology (Fig. 1; Table S3). Only the youngest nodes were consistently inferred correctly. Mouse and rat were always inferred as sister species, and the relationships among primates were correct in all 20 trees. The relationships between cow, horse, and dog were only correct for two sets of parameters (100 bp reads, clustering similarity = 90%, min. taxa = 4 and 6) but it is important to note that these relationships are not well supported by bootstrapping in the reference phylogeny [20].

For yeasts, only four of 20 parameter sets yielded completely accurate topologies (Fig. 1; Table S4): read length = 100 bp, clustering similarity = 60% or 70%, and min. taxa = 4 or 5. Relationships of the three most closely related taxa (*S. cerevisiae, S. paradoxus, S. mikatae*) were correctly reconstructed (*S. cerevisiae* and *S. paradoxus* sister to *S. mikatae*) for all parameter combinations with read length = 100 bp.

### Species tree estimation for *Drosophila*

The large number of loci, and limits of computer memory, precluded *BEAST analyses for many parameter combinations. In our tests, an 8-core Mac Pro with 8 GB of RAM could run *BEAST on datasets with up to approximately 700 loci. This included all parameter combinations where min. taxa = 9 (3–89 loci: 300–3706 bp). The largest of these matrices (100 bp reads, restriction enzyme = *SbfI*, clustering similarity = 60%) had 9,457 characters and ran in 4.2 hours. In contrast, the largest matrix used for maximum likelihood analysis (100 bp reads, restriction enzyme = *SbfI*, min. taxa = 4, clustering similarity = 55%) had almost 300,000 characters and the analysis was completed in 1.2 hours. Some *BEAST runs yielded 50% maximum clade credibility tree topologies that were different than the topologies from the concatenated analyses based on the same data, but overall accuracy was comparable, as measured by the number of correct nodes inferred with posterior support >70% (Table S2). The topological accuracy of *BEAST results differed from that of concatenated analyses by more than two nodes for only two sets of parameters (50 bp reads, restriction enzyme = *SbfI*, min. taxa = 9, clustering similarity = 80%; and 100 bp reads, restriction enzyme = *SbfI*, min. taxa 9, clustering similarity = 50%). For two sets of parameters, *BEAST recovered the same topology as the concatenated analyses of the same data. For twelve sets of parameters, concatenated analyses yielded trees that each had at least one more correct node than the corresponding *BEAST tree, while the converse was true for five sets of parameters. The differences in accuracy between the *BEAST and total evidence phylogenies were not strongly associated with particular sets of parameters.

## Discussion

### Orthology estimation

Without a reference genome, the most substantial obstacle to using RAD sequences for phylogenetics is determining orthology. We show here that clustering by sequence similarity is generally effective at grouping orthologous sequences (Figs. 2, S1, S2, S3; Table S2). Higher values of similarity increased the proportion of orthologous clusters in filtered datasets but reduced the total amount of data and the number of informative sites. *Drosophila* trees reconstructed from exclusively orthologous RAD sequences almost always matched the reference topology (Fig. 1), which suggests that in the absence of orthology estimation errors, RAD sequences contain useful phylogenetic signal. However, it also seems that errors in orthology estimation are not the primary cause of phylogenetic inaccuracy in our workflow. In many cases, even when the proportion of completely orthologous clusters was very low, the correct topology was inferred with moderate to high support (Fig. 3; Table S2). Clusters that are not completely orthologous often contain substantial amounts of phylogenetic signal, as shown by the moderate to high accuracy of trees reconstructed from only non-orthologous clusters, i.e., clusters that contain at least one non-orthologous sequence relative to the other sequences (Table S2).

The performance of clustering at recovering orthologous sequences is non-random with respect to phylogeny, and degrades with evolutionary divergence time. Deep divergences are problematic for two reasons: first, restriction sites change over time, with losses favored over gains, leading to a reduction in the number of orthologs retained across divergent taxa; second, evolutionary divergence of orthologous RAD sequences compromises the ability to infer their orthology based on sequence similarity. Consequently, taxa that are phylogenetically isolated on long branches are less likely to retain orthologous restriction sites, and the RAD sequences they do retain will be more divergent, diminishing their representation in clusters. Species of *Drosophila* without close relatives in our analysis (*D. ananassae*, *D. grimshawi*, *D. mojavensis*, *D. persimilis*, *D. pseudoobscura*, *D. virilis*, and *D. willistoni*) were represented in fewer clusters compared with the others (Fig. 2, S1, S2, S3). This systematic pattern to missing data is an important potential source of error in the design of phylogenetic studies using RAD sequences, and should be considered in the interpretation of inferred trees.

### Accuracy and clade age

Our workflow of clustering, filtering, and concatenation (Table 2) was able to accurately infer phylogenetic relationships for *Drosophila*, but was less successful for yeasts and mammals, although within each of these groups the phylogenetic relationships of closely related taxa were often accurate. This suggests that clade age and divergence times of lineages are important determinants of the success of the RAD method. *Saccharomyces* and *Candida* diverged approximately 300 million years ago (Mya) [24]. Among mammals, the Euarchontoglires (primates+rodents) diverged from the Laurasiatheria (cow+horse+dog) approximately 100 Mya [25]. By contrast, the crown age of the *Drosophila* species analyzed here is 40–60 Mya [26]–[28].

**Table 2:** A workflow for phylogenetic inference using RAD sequences.

Steps to determine if the species your wish to study are appropriate for RAD phylogenetics
How much evolutionary divergence time do you expect between taxa? RAD appears to work well for ≤50 million year divergences by consistently fails at ≥100 million years.
Collect samples
High quality genomic DNA is the required input. It is better if there is a continuum of relatedness between taxa so that each species has at least some close relatives included in the analysis.
Prep and sequence DNA
This can either be done in house or by sending samples to a sequencing facility [8]. The basic procedure is to cut genomic DNA with the specified restriction enzyme, randomly fragment the resulting pieces, barcode the samples for increased cost-efficiency, and sequence.
Filter sequences and call consensus loci
Som sequence reads will be ambiguous or of low quality. These should be discarded. High coverage of loci allows for probabilistic analyses of the most likely base at each position [12]–[15].
Cluster sequences (Step 1 from Methods)
A variety of clustering similarities should be tried to test the consistency and believability of results. UCLUST [16] is fast and effective at finding homologous sequences.
Choose minimum taxa cluster sizes (Step 2)
Small minimum taxa cluster sizes tend to produce the best topologies but larger values may be useful with very large datasets. Any cluster smaller than the chosen minimum taxa cluster size is excluded as are clusters with samples represented by multiple sequences.
Align clusters of sequences (Step 3)
Each cluster of sequences should be individually aligned using an automated alignment program. The volume of data precludes manual alignment.
Concatenate clusters (Step 3)
All clusters should be concatenated, filling in missing sequences from each cluster with gaps. There will be many missing sequences.
Reconstruct phylogeny
RAxML [19] is fast, can handle matrices even millions of base pairs long and can reconstruct accurate topologies from this type of data but other methods can be used.
Compare results from different parameters
Different sets of reasonably chosen parameters should produce similar topologies. Although low clustering similarities were successful in our study, higher similarities may be more useful for more recently divergent taxa. Low clustering thresholds may allow for more data, but more data may also be discarded if multiple sequences from a single species more often end up clustering together.

As noted previously, deep divergences reduce the amount of recoverable RAD loci between taxa. We consistently recovered accurate relationships within younger groups (≤40–60 Mya) including *Drosophila* over a broad range of matrix assembly parameters. In mammals, these clades included primates (approximately 22 Mya; [29]), and *Mus*+*Rattus* (approximately 10–30 Mya; [30], [31]). In yeasts, longer read lengths consistently yielded accurate relationships of the more recently diverged species (*S. cerevisiae*+*S. paradoxus* sister to *S. mikatae*), and correctly recovered the entire tree at low values of clustering similarity (60% and 70%). It appears that with longer read lengths, deeper divergences may be tractable with RAD sequences. The performance of RAD sequencing for phylogenetics is thus likely to improve as high-throughput sequencing technologies advance and yield longer reads.

### Phylogenetic signal and gene tree incongruence in *Drosophila*

We judged phylogenetic accuracy by comparing the inferred phylogenies to a single reference topology for each clade. However, within *Drosophila*, incomplete lineage sorting has led to widespread gene tree incongruence in the relationships between *D. erecta*, *D. yakuba*, and the *D. melanogaster* clade (*D. melanogaster* sister to *D. sechellia*+*D. simulans*; [32]). In the maximum likelihood *Drosophila* phylogeny (the one used as the reference here), *D. yakuba*+*D. erecta* form a clade sister to the *D. melanogaster* clade but this topology is not universally supported. Pollard and colleagues [32] analyzed more than 380,000 informative nucleotide changes in over 9,400 genes in the *Drosophila* genomes, and found that 55.3% of these changes support trees which differ from the reference topology in having either *D. yakuba* sister to *D. erecta*+the *melanogaster* clade, or *D. erecta* sister to *D. yakuba*+the *melanogaster* clade (Fig. 1). Similarly, 42.2% of genes support these alternative topologies with higher likelihood than the reference topology. The presence of one of these alternative nodes in the reconstructed trees is shown in Table S2.

We surmise that gene tree incongruence in the *Drosophila* genomes had a significant effect on our total-evidence reconstructions of phylogeny. Of the 572 phylogenies inferred across the range of clustering and filtering parameter values, 195 (34%) exactly matched the reference topology, implying that the remaining 377 phylogenies were incorrect. However, of these 377, 119 (32%) matched one of the two likely alternative topologies [32]. Overall, 314 (55%) of the total inferred phylogenies exactly matched one of the three topologies that are each supported by large portions of the genome. Of the 13 parameter combinations that produced the same topology across input-order replicates, 12 recovered either the perfect topology or one of the alternative topologies. Consistency across input-order replicates may therefore be a useful way of choosing parameter sets when analyzing real world RAD data.

The influence of gene tree incongruence on our ability to infer the *Drosophila* phylogeny shows that even these vast quantities of data can yield misleading results. RAD data are not resistant to the problems typically encountered in genome level molecular phylogenetics [33]. These potential pitfalls should be carefully considered when interpreting results.

### Missing data, accuracy, and support

In this study we had the benefit of reference phylogenies, against which we could compare the trees inferred from different combinations of data matrix assembly parameters. In practice, however, reference topologies are unlikely to be generally available. In that case, how much confidence should one have in a tree inferred from RAD sequences? Our results show that, while correct nodes are more likely in general to be strongly supported, incorrect nodes can also have high bootstrap values, although this is not unique to RAD phylogenetics [34]–[36]. This suggests that bootstrap support alone is not a sufficient measure of confidence, and consideration should be given to other factors, such as consistency across input-order replicates. Our success at accurately inferring phylogenies from data matrices with vast amounts of missing data suggests that missing data *per se* is not problematic for reconstructing phylogenies; rather, inaccuracy may arise more from phylogenetically misleading data and lack of informative data [33], [37]–[40]. Taxa with the fewest informative characters were more frequently placed incorrectly in the phylogeny, suggesting that the distribution of data across taxa is an important consideration. Moreover, our preliminary investigations indicate that for RAD matrices, the ratio of parsimony compatible sites to incompatible sites at each node may provide useful measures of confidence in tree topology. This also explains the trend towards more accurate trees when more data are available.

### Species tree estimation

Perhaps the greatest advantage of using RAD sequences for phylogeny reconstruction, as opposed to the traditional approach of using one to several genes, is that the RAD method samples data from many loci across the entire genome. This suggests that RAD sequence alignments could be profitably applied to methods for multilocus species tree estimation, an active area of current research that emphasizes the use of coalescent models to account for gene tree incongruence [41], [42]. Unfortunately, many multilocus methods (e.g., BUCKy; [21]) currently require complete data matrices, i.e., that all loci have been sequenced for all taxa. Interspecific RAD data sets do not fit this criterion, for reasons discussed previously. Other methods for reconstructing species trees from gene trees are prohibitively slow on large data sets (BEST; [43]), or have not been tested on data sets consisting of large amounts of missing data (*BEAST, STEM; [22], [23]). Our experiments running *BEAST with the *Drosophila* data met with limited success, but nevertheless held some hope about the prospects of species tree inference from RAD sequences. These inferences would potentially be more successful with the inclusion of multiple individuals per species, allowing for more useful analyses of coalescence. As with the concatenation approach, *BEAST trees were most accurate when based on long sequence read lengths. This suggests that as second generation sequencing methods begin to produce longer reads, the accuracy of using the RAD method for phylogenetics will improve.

The future of species tree estimation from RAD sequences likely does not lie in current methods that are based on coalescence and reconciliation of gene trees. Rather, it may be necessary to design phylogenetic inference methods specifically for RAD sequencing, due to the fact that individual RAD loci generally do not have sufficient variation to reconstruct completely resolved gene trees, and are expected to have phylogenetically structured patterns of missing data. Bryant and colleagues [44] describe a promising method for estimating phylogenetic trees from SNP and AFLP (presence-absence) data that is being implemented in the development version of BEAST. Continued development of these methods will surely increase the usefulness of RAD sequencing for phylogeny estimation.

### RAD sequencing in practice

Our analyses of *Drosophila* reveal some general patterns in how sequence read length, clustering and filtering parameters influence phylogenetic accuracy. As might be expected, longer sequences perform better. We found that values of clustering similarity in the range of 55–70% generally produced the most accurate topologies, possibly because these produced matrices with more informative sites than at higher values of clustering similarity. However, this result may be taxon-specific and exploring a range of clustering similarity values is generally advisable. If UCLUST is used for clustering, we also suggest conducting replicate analyses in which the input order of sequences is randomized. Consistency across large numbers of replicates may indicate appropriate parameter values (Table S6). Regarding filtering for minimum number of taxa in a cluster, there appears to be little reason to use any value other than four, as higher proportions of missing data did not adversely affect the accuracy of inferred trees; an exception to this might arise if computer memory was limited, and smaller matrices were desired for that reason (e.g., for *BEAST analyses).

Our finding that topological accuracy is generally high across a wide range of clustering and filtering parameters suggests that phylogenetic inference using RAD data should be robust to a variety of read lengths, restriction enzymes, minimum cluster sizes, and clustering similarity values. Phylogenetic consistency across replicates of particular parameters tends to indicate that the resulting topology is correct. Our experiments used restriction enzymes that produced relatively few reads, but less selective enzymes would produce larger amounts of data that could further increase the quality and robustness of phylogenetic inference. In general, longer and more GC-rich restriction sites will be more conserved across taxa and will yield fewer RAD sequences compared with shorter restriction sites. Clearly, there is a more or less direct relationship between the number of loci that are sequenced and the performance of phylogenetic analysis. The availability of software tools such as UCLUST, which can rapidly cluster millions of sequences on a desktop computer, facilitates the management and analysis of large data sets.

We show that it is possible to use RAD sequence data to accurately reconstruct phylogenies, but our *in silico* experiments omitted several steps that would be required in applying the method in practice to a given set of non-model species. Most importantly, we did not consider the problem of generating consensus sequences of RAD loci for each taxon, which requires accounting for the error rate in Illumina sequencing (currently about 1%, increasing toward the ends of reads). Likelihood-based methods for this step have been developed (e.g. [12]–[15]). To date, RAD sequencing has not been done using the SOLiD platform, which has a lower error rate than Illumina [45].

It would be theoretically possible to use a longer read technology, such as 454, to obtain RAD data with more data per locus. Our preliminary analyses suggest that this increase in data does not significantly improve phylogenetic accuracy, but could help resolve the placement of taxa otherwise under-represented in the data matrix. However, large numbers of reads per sample are necessary to call consensus sequences for each locus, and the longer read technologies would require more runs to obtain the same coverage across many samples and loci. The comparatively high cost per sequence of longer read technologies make them ineffective for RAD phylogenetics at this point in time. Further developments in both sequencing technologies and computational tools will continue to improve the utility of RAD sequencing.

## Supporting Information

Figure S1 **As in** **Figure 2** **except showing a replicate of each matrix constructed from 50 bp reads and the restriction enzyme** ***SbfI*** **.**
(TIF)

Figure S2 **As in** **Figure 2** **except showing a replicate of each matrix constructed from 100 bp reads and the restriction enzyme** ***NotI*** **.**
(TIF)

Figure S3 **As in** **Figure 2** **except showing a replicate of each matrix constructed from 50 bp reads and the restriction enzyme** ***NotI*** **.**
(TIF)

Table S1 **Accuracy of** ***Drosophila*** **trees reconstructed from RAD data of known orthology and alignment.**
(XLS)

Table S2 **Data characteristics and phylogenetic accuracy of every combination of parameters used for** ***Drosophila*** **.**
(XLS)

Table S3 **Details of the data and the accuracy of the resulting trees for every supermatrix of mammal sequences.**
(XLS)

Table S4 **Details of the data and the accuracy of the resulting trees for every supermatrix of fungus sequences.**
(XLS)

Table S5 **Results of Mann-Whitney tests for differences in bootstrap support between correct and incorrect nodes.**
(XLS)

Table S6 **Phylogenetic accuracy for 100 randomized replicates of a subset of parameters.**
(XLS)
